# Acute Laceration in the Transverse Mesocolon After a Colonoscopy: A Case Report

**DOI:** 10.7759/cureus.67427

**Published:** 2024-08-21

**Authors:** Yongbo Zhang, Linchuan Li, Changhong Liu, Mingwen Jiao, Jingbo Chen

**Affiliations:** 1 Department of General Surgery, The First Affiliated Hospital of Shandong First Medical University, Jinan, CHN; 2 Department of Gastroenterology, The First Affiliated Hospital of Shandong First Medical University, Jinan, CHN

**Keywords:** laparoscopic exploration, hemoperitoneum, clinical case report, transverse mesocolon laceration, colonoscopy complication

## Abstract

Colonoscopy is a widely used examination for colonic diseases with low risk. Hemoperitoneum due to injury of transverse mesocolon is an extremely rare but potentially lethal complication. We present a case of an elderly woman who complained of continuous abdominal pain after a colonoscopy, with progressive anemia. An emergency exploratory laparoscopy revealed a laceration of the transverse mesocolon. The underlying mechanism is unclear due to its rarity. Old age, atherosclerotic disease, the long operating time of colonoscopy, and manual compression on the abdomen during the procedure may be risk factors for transverse mesenteric laceration during colonoscopy.

## Introduction

Colonoscopy is widely used as a method of diagnosis and intervention of colonic diseases in our country. Bleeding and perforation of the colon have been reported as the most common complications [1]. Laceration of the mesocolon is a rare but potentially lethal complication. To the best of our knowledge, only one case was reported in 1999 [2]. Here, we report another case of an old female patient who suffered an acute injury of the transverse mesocolon after a colonoscopy. We summarized several common characteristics of the two cases, trying to provide some warning information to gastroenterologists.

## Case presentation

An 84-year-old woman, who complained of lower abdominal pain for more than three months, accompanied with difficult defecation, was hospitalized. She had a history of left inguinal hernia for more than 30 years, hypertension for more than 20 years, and coronary heart disease for more than 10 years. She was taking metoprolol, amlodipine, and atorvastatin. No anticoagulant or antiaggregant drugs were used recently. Physical examination showed her BMI was 16.3 kg/m^2^. Mild tenderness was found in the lower abdomen, while rebound tenderness was negative. A diagnostic colonoscopy was carried out under propofol anesthesia after some routine examinations and mechanical bowel preparation. The colonoscope was advanced to the terminal ileum while gentle abdominal manual compression was applied to the left lower quadrant, which lasted approximately 30 minutes. Meanwhile, a single 8-mm polyp was snared in the transverse colon.

**Figure 1:**
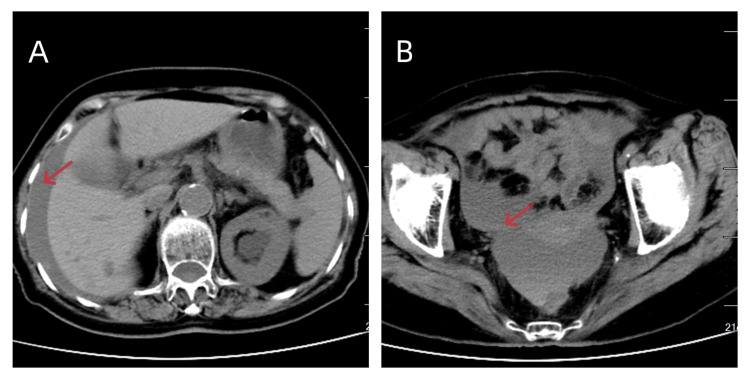
CT scan showed seroperitoneum in the perihepatic space (A) and pelvic cavity (B). No free air was noticed.

Right after recovery from anesthesia, she began to complain of continuous abdominal pain with progressive anemia. Her blood pressure was 118/88 mmHg and her pulse rate was 100/min. She exhibited diffuse abdominal tenderness with rebound tenderness. A blood routine test revealed hemoglobin decreased from 131 g/L to 109 g/L (the reference range for our laboratory is 115-150 g/L). A CT scan revealed a seroperitoneum with a density level consistent with blood, which was highly suspicious of hemoperitoneum (Figure 1). And there was no evidence of spleen or liver injury.

**Figure 2:**
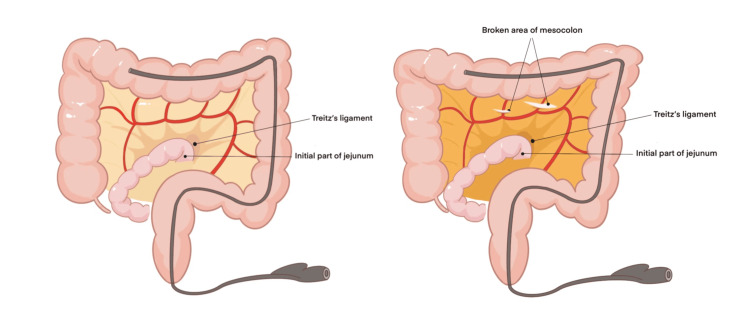
We drew a schematic diagram showing the findings of the surgical exploration. Two holes were verified in the transverse mesocolon during the laparoscopic exploration, which together with the collateral branches of mid-colic vessels was lacerated.

**Figure 3:**
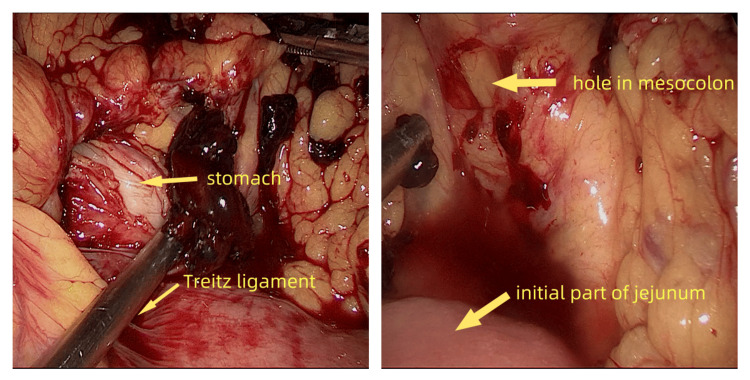
Two holes were found in the transverse mesocolon and branches of mid-colic vessels were bleeding.

An emergent laparoscopic exploration was performed promptly showing a large formation of blood in the peritoneal cavity. About 1500 ml of free blood fluid and some clots were aspirated from the peritoneal cavity. The patient required four units of leukoreduced red blood cells during the operation to maintain a hemoglobin above 100g/dl. After peritoneal irrigation with a large amount of normal saline, we found the liver and spleen had no traumatic lesions in appearance. There were no obvious macroscopic perforations or lesions on the stomach, small intestine, and colon. Two holes were verified in the transverse mesocolon (Figure 2), which together with the collateral branches of mid-colic vessels was lacerated. Bleeding was observed on the lacerated vessel stumps (Figure 3). Laceration of blood vessels led to severe intra-abdominal hemorrhage. Hemostasis was achieved using several Hem-o-lok polymeric clips. The patient recovered from surgery uneventfully and was discharged from the hospital eight days later.

## Discussion

In general, complications after a colonoscopy are rare. Among them, colonic bleeding and perforation are the two most common complications, with an incidence of 0.26% and 0.05%, respectively [1]. Intra-abdominal hemorrhage is a rare complication of colonoscopy that is attributable mostly to splenic injury [3]. Other causes of hemoperitoneum, such as laceration of the sigmoid colonic seromuscular [4], mesocolon of the descending colon [5], and rupture of ovarian stromal tumors [6], are extremely rare.

Because of the lower incidence of intra-abdominal bleeding compared with intra-luminal bleeding, sometimes the intra-abdominal bleeding might be obscured by concomitant intra-intestinal bleeding, leading to delayed diagnosis and increased morbidity for the patient [7]. In this situation, CT imaging can be a first-line imaging tool for differential diagnosis [8] in which blood has a higher density than water.

We present a case of transverse mesenteric laceration after colonoscopy. Laceration of the blood vessels resulted in blood loss and a decrease in circulatory blood volume. Thanks to the swift action of surgical treatment, two lacerated holes in the transverse mesocolon with actively bleeding vessels were noted. Thus, the bleeding was promptly stopped. However, the underlying mechanism of transverse mesenteric laceration is unclear. Here, we offer a hypothesis. To complete a colonoscopy, overcoming several anatomical angles is indispensable. Surgeons have to rotate, move, retract, and push the probe in and out many times. During the procedure, the colon together with its mesentery is affected, stretched, and even may be lacerated. Our patient, a slender woman with a BMI of 16.4 kg/m², likely had reduced fat content in her mesentery. Additionally, the patient had a history of coronary heart disease. Meanwhile, a CT scan showed calcifications in the abdominal aorta. It is reasonable to suspect that extensive atherosclerotic vascular calcifications, particularly in the mesenteric arteries, combined with reduced mesenteric fat content, predisposed the mesentery and vessels to laceration.

Another similar case was reported in 1999 [2], a 72-year-old man developed hemoperitoneum after a colonoscopy, confirmed to be a laceration in the transverse mesocolon. According to the literature published, the two cases had several common characteristics, such as old age, atherosclerotic disease, the long operating time of colonoscopy, and manual compression on the abdomen during the procedure, indicating that these may be risk factors of transverse mesenteric laceration. Fortunately, both patients received emergency operations timely, thus avoiding death.

## Conclusions

Hemoperitoneum is a rare complication. And hemoperitoneum due to laceration of mesocolon is extremely rare. We suggest that gastroenterologists should be aware of this life-threatening situation for every acute abdominal pain after colonoscopy. A careful observation period following colonoscopy is crucial, as well as appropriately informing patients of possible symptoms indicative of bleeding. Abdominal CT scans and blood routine tests are useful tools in this situation. Early diagnosis and appropriate treatment may improve the prognosis of the patient.
